# Right Ventricular Morphology in PA/IVS: Integrating Developmental Pathology With Echocardiographic Prognostication

**DOI:** 10.1111/echo.70190

**Published:** 2025-05-19

**Authors:** Ghassan Alnaami

**Affiliations:** ^1^ Family First Medical Clinic Edmonton Alberta Canada

## Commentary

The manuscript by Moras et al. [1] introduces a critical framework for individualized post‐interventional care in neonates with pulmonary atresia with intact ventricular septum (PA/IVS) or critical pulmonary stenosis (CPS) by leveraging echocardiographic classification of right ventricular (RV) morphology. Their work aligns directly with the developmental understanding of RV anatomy, as outlined in the next paragraph on RV developmental pathology.

**FIGURE 1:**
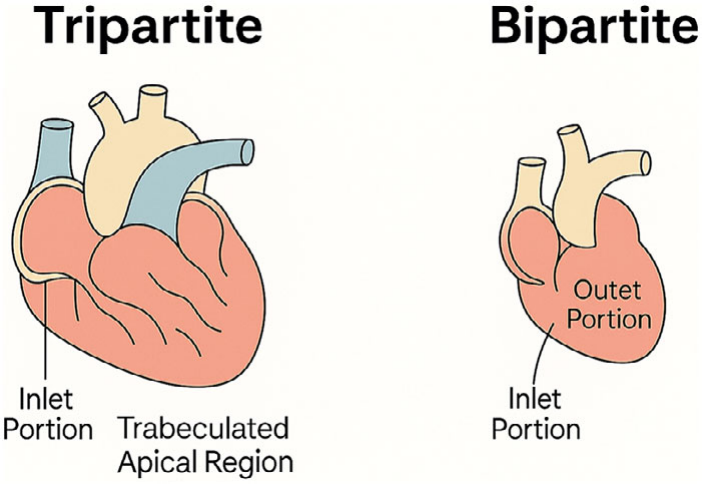
Bipartite RV is composed of only two parts: the inlet and a hypoplastic outlet in most cases.

## Developmental Basis of RV Morphology

The normal right ventricle is tripartite, composed of:

1. Inlet portion (receives blood via the tricuspid valve),
2. Apical trabeculated region (contributes to contractility),
3. Outlet or infundibular portion (directs flow to the pulmonary artery).

Failure of development or muscular obliteration of the apical and/or infundibular segments leads to bipartite or monopartite RVs. (Figure 1) These developmental anomalies underlie many presentations of congenital heart disease, including tricuspid or PA, and significantly influence the RV's ability to respond to decompression interventions (See Table 1)

**TABLE 1:** Embryological origins and functions of the right ventricular components.

Right ventricular region	Embryological origin	Structures included	Function
Inlet portion	Primitive ventricle of the early heart tube	Tricuspid valve, chordae tendineae, papillary muscles	Receives deoxygenated blood from the right atrium
Trabeculated apical region	Also from the primitive ventricle	Muscular trabeculations	Contributes to myocardial contractile force
Outlet (infundibulum/conus)	Bulbus cordis (conus cordis segment)	Smooth‐walled outflow tract to pulmonary valve and artery	Channels blood from RV into the pulmonary circulation

In their study, Moras et al. classify patients into two groups based on this anatomical distinction: Group A (tripartite RVs) and Group B (bipartite RVs). This classification strongly predicted complications post‐Pulmonary Valve Balloon Dilatation (PVBD): Tripartite RVs were associated with left ventricular (LV) systolic dysfunction due to volume redistribution and interventricular interaction. Bipartite RVs were prone to infundibular spasm, evidenced by dynamic outflow obstruction requiring beta‐blockade.

## Clinical Implications of Morphotype Stratification

The clinical implications of this work are profound. Moras et al. demonstrate that 92.9% of patients with tripartite RVs experienced transient LV dysfunction, necessitating inodilator therapy and prolonged ventilation. In contrast, 76.9% of bipartite RV patients exhibited infundibular spasm managed effectively with beta‐blockers.

This distinction supports the development of a morphotype‐specific post‐procedural algorithm:

· **Tripartite RVs**: Anticipate LV dysfunction, initiate inodilators early, delay extubation, and monitor for mesenteric ischemia before initiating enteral feeds.
· **Bipartite RVs**: Monitor for dynamic RV outflow tract obstruction, administer beta‐blockers, and assess the need for additional pulmonary blood flow support.

In tripartite RVs, the left ventricle often encounters a sudden increase in preload following decompression due to restored antegrade pulmonary flow. This surge in pulmonary venous return, compounded by ventricular interdependence and an under‐conditioned LV, can result in transient systolic dysfunction. The LV may be particularly susceptible if it was relatively underloaded in utero. These physiologic shifts necessitate early initiation of inodilator therapy, cautious fluid management, and delayed enteral feeding until hemodynamic stability is achieved [2].

By individualizing care based on early echocardiographic findings, outcomes may improve, and unnecessary interventions may be avoided.

## Surgical Versus Transcatheter Approach: A Developmental Consideration

In addition to phenotype‐specific care, our knowledge of RV developmental pathology adds valuable context comparing transcatheter RV decompression with open‐heart surgical strategies. Although transcatheter approaches offer minimally invasive access with the potential to promote RV growth, high reintervention rates and unsuitability for RV‐dependent coronary circulation limit their use in certain morphologies. Open‐heart surgery remains a viable, though more invasive, option with potential for definitive repair in select cases, typically RV‐dependent coronary circulation and non‐trabeculated or monopartite RV [3].

This dichotomy further reinforces the need for developmental and anatomical considerations in early decision‐making. Understanding which RV segments are present prenatally or at birth may help anticipate whether a patient can achieve a biventricular repair or is destined for single‐ventricle palliation.

The contribution by Moras et al. elegantly merges echocardiography with developmental anatomy to guide real‐time neonatal care. As our understanding of RV morphogenesis advances, so too must our strategies to support these vulnerable infants.

The work prompts further research avenues:

1. Can prenatal echocardiographic imaging predict RV phenotype and inform delivery planning?
2. Can machine learning models use echocardiographic data to automate phenotype classification and predict ICU trajectories?
3. Can RV function modalities (strain, speckle tracking, TAPSE) be applied to fetal echocardiographic studies to be integrated into therapeutic planning?

These extensions could lead to a new era of precision neonatal cardiology—one grounded in the biology of development and expressed through the lens of bedside diagnostics (See Table 2)

**TABLE 2:** Right ventricle function echocardiography tools that can be used pre‐ and postnatal.

Parameter	Function measured	Advantages	Limitations
TAPSE	Longitudinal systolic	Simple, reproducible	Angle and load‐dependent
TDI S'	Longitudinal systolic	Early dysfunction detection	Load‐ and angle‐dependent
FAC	Global systolic	Quantitative, standard cutoff	Requires a clear endocardial definition
RIMP (Tei index)	Global (systolic + diastolic)	Independent of shape	Affected by heart rate, rhythm
STE (strain)	Myocardial deformation	Detects subclinical dysfunction	Vendor differences, limited availability
3D echo	Volume, ejection fraction	Most accurate RV EF	Image quality, technical demand

Abbreviations: 3D echo, three‐dimensional echocardiography; FAC, fractional area change; RIMP (Tei index), right ventricular index of myocardial performance; STE (strain), speckle‐tracking echocardiography (longitudinal strain); TAPSE, tricuspid annular plane systolic excursion; TDI S', tissue doppler imaging, systolic velocity at the tricuspid annulus.

Lastly, here is a table summarizing various echocardiographic methods to measure the RV function, which can be used in congenital heart disease, too. Unlike postnatal imaging, fetal echocardiography faces unique challenges, including limited views, high fetal heart rate, and absence of standardized RV pressure/volume measurements [4].

## Conflicts of Interest

The author declares no conflicts of interest.

## Data Availability

The data that support the findings of this manuscript are available in PubMed. Detailed information is provided in the reference section.
